# Photocleavage-based affinity purification of biomarkers from serum: Application to multiplex allergy testing

**DOI:** 10.1371/journal.pone.0191987

**Published:** 2018-02-01

**Authors:** Zhi Wan, Heather P. Ostendorff, Ziying Liu, Lynda C. Schneider, Kenneth J. Rothschild, Mark J. Lim

**Affiliations:** 1 AmberGen, Incorporated, Watertown, Massachusetts, United States of America; 2 Department of Pediatrics, Boston Children’s Hospital, Boston, Massachusetts, United States of America; 3 Molecular Biophysics Laboratory, Department of Physics and Photonics Center, Boston University, Boston, Massachusetts, United States of America; Chang Gung University, TAIWAN

## Abstract

Multiplex serological immunoassays, such as implemented on microarray or microsphere-based platforms, provide greater information content and higher throughput, while lowering the cost and blood volume required. These features are particularly attractive in pediatric food allergy testing to facilitate high throughput multi-allergen analysis from finger- or heel-stick collected blood. However, the miniaturization and microfluidics necessary for creating multiplex assays make them highly susceptible to the “matrix effect” caused by interference from non-target agents in serum and other biofluids. Such interference can result in lower sensitivity, specificity, reproducibility and quantitative accuracy. These problems have in large part prevented wide-spread implementation of multiplex immunoassays in clinical laboratories. We report the development of a novel method to eliminate the matrix effect by utilizing photocleavable capture antibodies to purify and concentrate blood-based biomarkers (a process termed PC-PURE) prior to detection in a multiplex immunoassay. To evaluate this approach, it was applied to blood-based allergy testing. Patient total IgE was purified and enriched using PC-PURE followed by multiplex microsphere-based detection of allergen-specific IgEs (termed the AllerBead assay). AllerBead was formatted to detect the eight most common pediatric food allergens: milk, soy, wheat, egg, peanuts, tree nuts, fin fish and shellfish, which account for >90% of all pediatric food allergies. 205 serum samples obtained from Boston Children’s Hospital were evaluated. When PC-PURE was employed with AllerBead, excellent agreement was obtained with the standard, non-multiplex, ImmunoCAP^®^ assay (average sensitivity above published negative predictive cutoffs = 96% and average Pearson r = 0.90; average specificity = 97%). In contrast, poor ImmunoCAP^®^-correlation was observed when PC-PURE was not utilized (average sensitivity above published negative predictive cutoffs = 59% and average Pearson r = 0.61; average specificity = 97%). This approach should be adaptable to improve a wide range of multiplex immunoassays such as in cancer, infectious disease and autoimmune disease.

## Introduction

### Multiplex assays and the problem of the matrix effect

A major problem for biomarker-based diagnostic assays is the low sensitivity and specificity provided by a single biomarker. To overcome this problem, researchers have focused on developing effective multi-biomarker panels. For example, blood-based panels consisting of three or more protein biomarkers have been reported for a variety of cancers [1–6] and autoimmune diseases [7, 8]. As discussed later, blood-based allergy testing is also highly amenable to multi-biomarker analysis [9–12].

A high priority is now to transition these multi-biomarker panels to multiplex assay formats both for large-scale biomarker validation and ultimately for clinical assay. In this regard, the utilization of multiplex platforms will be critical for providing lower cost and higher throughput, especially necessary for population-wide screening. Furthermore, the amount of blood available for multiple biomarker testing is limited. One example is the case of clinical trials where a sample may be divided up for many different purposes. A second example is blood drawn using finger-stick and heel-stick methods for pediatric diagnostic testing where only ~100–200 μL of serum is typically available [13, 14].

A variety of solid-phase immunoassay platforms have been developed to meet the needs for multiplex analysis. Mainstream platforms include those based on microarrays, such as the MSD MULTI-ARRAY^®^ technology [15], and coded microspheres, such as the Luminex^®^ xMAP^®^ platform [16]. Although these systems have been useful for basic research, they have generally failed to transition into the clinical diagnostic laboratory [17].

**Fig 1:**
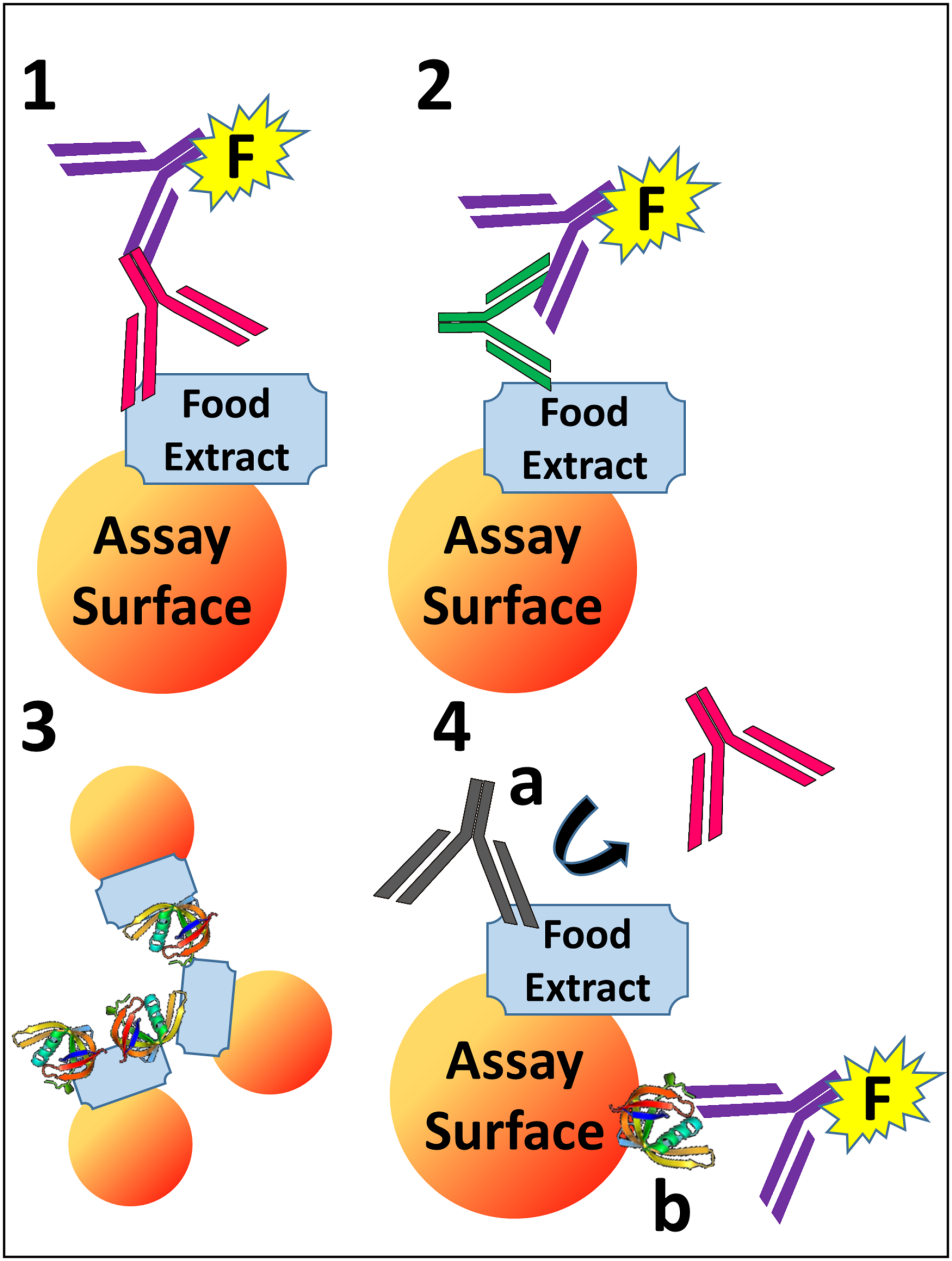
Diagrammatic representation of the matrix effects in multiplex immunoassays: Example of food allergy testing. Y-shaped structures are antibodies and are as follows: Red = Patient Allergen-Specific IgE (sIgE); Purple = Detection Antibody; Green = Heterophile Antibody; Gray = Non-IgE Allergen-Specific Immunoglobulin. (1) Normal configuration of a multiplex Luminex^®^ assay with a whole food extract (light blue) as the “bait” antigen(s) on the coded microsphere (“Assay Surface”) with bound patient IgE (red) and detection antibodies (purple with fluorescent label “F”). (2) Low-specificity heterophile antibodies (green) in human blood can bridge the detection antibody to proteins (*e*.*g*. non-immune globulins or immunoglobulins) on the microsphere, yielding a false positive signal. (3) Matrix-induced microsphere aggregation can also occur (*e*.*g*. caused by specifically or non-specifically bound unintended matrix constituents). (4) Non-specific or even specific binding of any unintended matrix constituents to any component of the immunoassay can interfere, for example by (a) blocking IgE binding (*e*.*g*. due to a competitively bound non-IgE allergen-specific immunoglobulin) or (b) mediating background signals.

A major limitation of most multiplex assays, such as those based on the Luminex^®^ xMAP^®^ system, is the well-known “matrix effect” [18–25]. This effect is caused by the presence of non-target constituents in blood such as proteins, cholesterol, lipids, salts and low-specificity heterophile antibodies which interfere with detection of the often less abundant biomarkers. While all assays are impaired by the matrix effect to some degree, the effect is exacerbated in multiplex assays mainly because of the lower binding capacity of the miniaturized assay surfaces (*e*.*g*. microspheres and microarrays). Such surfaces quickly saturate with the specific or non-specific binding of unintended matrix components from the sample (which can suppress signal, mediate background and/or cause microsphere aggregation; Fig 1). In addition, high viscosity and undesirable conductance of the sample matrix can interfere with microfluidics [26, 27], which are commonly used in today’s multiplex assay platforms.

In one example of the matrix effect, a recent study by Rosenberg-Hasson et al. of multiplex Luminex^®^ assays for 51 cytokines [28] found that serum and plasma induced signal suppression up to 100-fold as well as non-linearity of dilution. In a second study Dias et al. found, using a multiplex assay for HPV on the Luminex^®^ platform, that it was often necessary to use minimally diluted or undiluted samples to detect the low abundance biomarker [29]. However, this is where the matrix effect is most severe. They also point out that the matrix effect varies by patient [29], not only affecting sensitivity, but also reproducibility and quantitative accuracy.

### Eliminating the matrix effect: Application to allergy testing

Current assessments show that 5–8% of all children in the U.S. have food allergies with 40% having a history of severe and potentially dangerous reactions [30, 31]. Furthermore, the prevalence of food allergies is rapidly increasing. According to a 2009 report from the Centers for Disease Control (CDC), food allergy prevalence in children increased 18% from 1997 to 2007, with resultant ER visits tripling in that period [30, 32]. Reported cases of nut allergies also tripled in a similar period [33]. In addition, there was an approximate doubling of hospital discharges in the US for diagnosis of food allergy between 1998 and 2006 as reported by the CDC [30].

Blood-based allergy testing forms an important part of the diagnostic and disease monitoring regimen [30, 31, 34]. The current standard approach is the ImmunoCAP^®^ system (Phadia/Thermo Fisher Scientific), which measures the concentration of circulating allergen-specific IgE antibodies (sIgE) in human serum or plasma (by measuring sIgE binding to allergen immobilized on an assay surface, using a fluorescence enzyme immunoassay [FEIA] format). ImmunoCAP^®^ offers quantitative sIgE measurements from 0.10 kIU_A_/L to 100 kIU_A_/L.

However, such tests require large blood volumes in order to measure each allergen one at a time. Therefore, these “single-plex” methods are not ideally suited for current and emerging pediatric food allergy testing needs. In contrast, multiplex assays have the potential to meet these needs, which include the following: Multi-Allergen Testing—At initial diagnosis, when a patient’s allergies are unknown, it may be necessary to test for multiple suspected foods, especially since a third of allergic children react to more than one food [31]. Furthermore, testing against purified allergen component proteins in addition to conventional whole food extracts is emerging as a useful clinical tool, for example in predicting severe allergy and anaphylaxis [35, 36]. Low Blood Volume Testing—While the need is growing to test patients for multiple allergens, this need is particularly problematic in infants/children where it is often difficult to collect sufficient blood volume to perform these multiple tests. For example, venipuncture from small children is difficult to accomplish and normally requires a highly skilled phlebotomist. Risks include bleeding and/or bruising, injury from restraining of the child, discomfort and mental stress for both the child and parents [37]. Low blood volume collection such as through a finger- or heel-stick is especially important for pediatric patients. High Throughput Screening—A recent landmark study (LEAP) [38–40] sponsored by the National Institute of Allergy and Infectious Diseases (NIAID) concluded that, contrary to many well-accepted guidelines, there was an overall 81% reduction of peanut allergy in at-risk children who began continuous consumption of peanuts at infancy, compared to those who avoided peanuts. Indeed, recent updated NIAID guidelines [41] recommend screening at-risk infants for peanut sensitization prior to engaging in early consumption therapies, to prevent serious adverse reactions which can occur due to allergen sensitization even *in utero* or during breastfeeding [42, 43]. This early-consumption benefit may potentially apply to other highly allergenic foods [44, 45].

**Fig 2:**
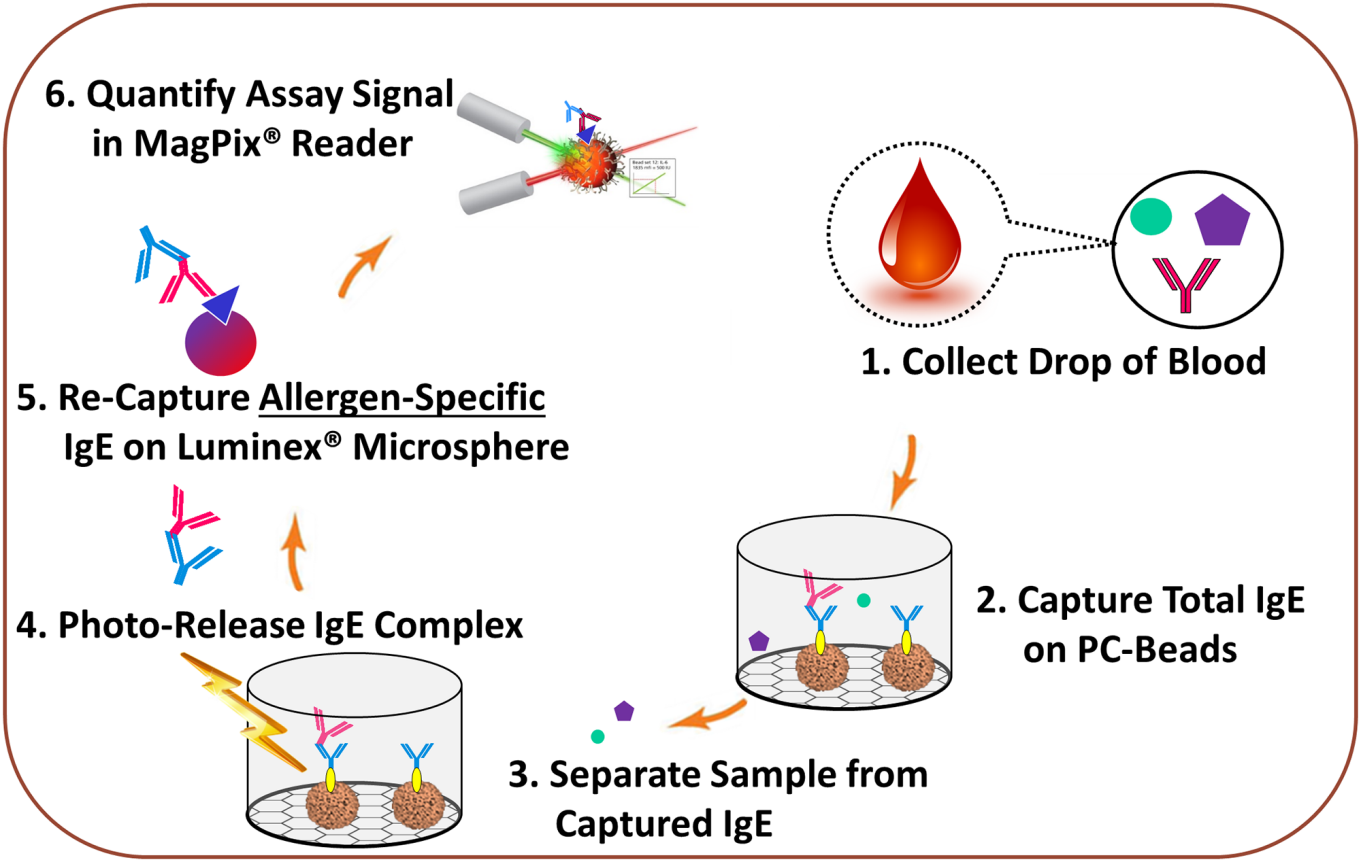
Eliminating the matrix effect with photocleavage based affinity purification of biomarkers (PC-PURE): Application to a downstream multiplex in vitro allergy assay (AllerBead). The biomarker, serum IgE in this case, is pre-purified using photocleavable antibodies (PC-Antibodies) attached to beads (PC-Beads), in an overall process termed PC-PURE. This eliminates interference from sample matrix constituents in the downstream multiplex immunoassay. The multiplex immunoassay shown here is based on Luminex^®^ coded microspheres which contain the “bait” allergen (antigen) for binding and quantifying sIgE (termed the AllerBead assay). Y-shaped structures are antibodies. The red antibody is the patient’s total IgE and blue is the anti-IgE PC-Antibody used for PC-PURE. The yellow oval is the photocleavable linker that attaches the PC-Antibody to high capacity agarose beads to form the PC-Beads. The processing of the PC-Beads is performed in microtiter filter plates. Following PC-PURE, the photocleaved PC-Antibody can be used also for detection in the AllerBead assay, or, a separate detection antibody (binding a different epitope) can be used, as was the case in this work (not depicted). The blue triangle at Step 5 represents the allergen(s) (antigens) immobilized on the Luminex^®^ assay surface (the coded microsphere).

Therefore, while multiplex assays can meet these needs, they must maintain the same level of sensitivity, specificity and quantitative accuracy compared to their non-multiplex, gold-standard counterparts, yet remain hindered by the matrix effect. To overcome this problem, we have developed a new approach, termed PC-PURE, which uses photocleavable antibodies (PC-Antibodies) to purify and concentrate patient total IgE prior to input into a multiplex sIgE immunoassay (diagrammatically shown in Fig 2). We have previously shown that PC-Antibodies could effectively capture and purify expressed proteins for proteomic applications [46]. Here, we report the application of PC-PURE for pre-purification of patient IgE for improved multiplex blood-based food allergy testing.

## Materials and methods

### Supplies and reagents

3-Amino-3-Deoxydigoxigenin Hemisuccinamide Succinimidyl Ester was purchased from Thermo Fisher Scientific (Waltham, MA). Purified human IgE, the IgG Mouse ELISA Kit and the Immunoglobulin IgE Human ELISA Kit were from Abcam (Cambridge, MA). Mouse monoclonal anti-human IgE antibodies (Clones E411 and 4F4cc) were from HyTest (Turku, Finland). PD SpinTrap G-25 Columns were from GE Healthcare Life Sciences (Pittsburgh, PA). Carboxyl-terminated MagPlex^®^ magnetic microspheres were from Luminex^®^ Corporation (Austin, TX). A mouse monoclonal anti-Digoxigenin antibody (Clone 1.71.256) and the purified natural allergen component protein lactalbumin (Bos d 4) were purchased from Sigma-Aldrich (St. Louis, MO). All other allergen component proteins were purchased from Indoor Biotechnologies (Charlottesville, VA). Whole food extracts were from Allergy Laboratories, Inc. (Oklahoma City, OK) and from the Research Department at Greer Allergy Immunotherapy (Lenoir, NC).

### Preparation of a digoxigenin labeled IgE (Dig-IgE) tracer

Commercially available purified human IgE as supplied was supplemented to 100 mM sodium bicarbonate from a 1M stock. A 10-fold molar excess of 3-Amino-3-Deoxydigoxigenin Hemisuccinamide Succinimidyl Ester labeling reagent was added from a 1 mM stock in DMSO. The reaction was carried out for 30 min with gentle mixing, protected from light. The reaction was then quenched by adding 1/9^th^ volume of 1 M glycine and subsequently mixing for 15 min. To avoid losses in the subsequent desalting column, a BSA carrier was then added from a 10% (w/v) stock to yield a final 0.05% (w/v). To remove unreacted labeling reagent, the reaction mix was then desalted on PD SpinTrap G-25 columns. The PD SpinTrap G-25 columns were performed according to the manufacturer’s instructions (equilibration in 300 μL of TBS). Following desalting, the final product corresponding to the digoxigenin labeled human IgE (Dig-IgE) was supplemented with 1/9^th^ volume of 10X TBS before aliquoting and storing at -70°C. The yield of Dig-IgE was quantified using the commercial Immunoglobulin IgE Human ELISA Kit.

### PC-Beads for IgE concentration and/or purification (PC-PURE) followed by multiplex sIgE immunoassay

Labeling of an antibody with PC-Biotin, to create the PC-Antibody, and attachment to streptavidin agarose beads, to create the PC-Beads, was performed as described previously [46]. The PC-Antibody, in this case an anti-IgE antibody (Clone E411), was loaded at a level of 5 μg per each 1 μL of packed bead pellet volume to form the PC-Beads. Processing of the PC-Beads for IgE concentration and/or purification was done in 96-well microtiter filter plates using a vacuum manifold, unless otherwise specified. 5 μL bead pellet volume of PC-Beads per well was washed briefly 4x 200 μL with TBS-T followed by the addition of 100–500 μL (see Results and discussion for specific volumes) of Dig-IgE containing sample or undiluted serum sample (discarded and de-identified serum samples were obtained from Boston Children’s Hospital under approval from their IRB; note that because the samples were serum samples obtained from subjects presenting at Boston Children’s Hospital for medical reasons completely unrelated to and independent of this study, informed consent was waived by the IRB; analysis of the samples at AmberGen was approved by AmberGen’s IRB, New England IRB [NEIRB], Needham, MA). PC-Beads and sample were mixed together for 1 hr to capture total IgE, followed by washing the PC-Beads 4x 200 μL briefly and 3x 200 μL for 10 min each (with mixing) in TBS-T. Photo-release in 100 μL BSA Block (1% BSA [w/v] in TBS-T) was achieved directly in the filter plate by illuminating as previously described [46] (note that 365 nm light at 7 mW/cm^2^ was delivered for 20 min using an ELC-500 UV Cure Chamber, Fusionet, LLC, Limington, Maine). The filter plate was then mixed, in the presence of the MagPlex^®^ allergen-coated microspheres, for 30 min (note that allergens were coated onto Carboxyl-terminated MagPlex^®^ magnetic microspheres using standard EDC/NHS chemistry according to instructions provided by the microsphere manufacturer for protein attachment [Luminex^®^ Corporation, Austin, TX]). The MagPlex^®^ microspheres were magnetically separated from the filter plate (non-magnetic PC-Beads remain behind) into a deep-well microtiter plate and washed briefly 3x 900 μL with TBS-T. Microspheres were then probed for 30 min with mixing using 100 μL/well of 1 μg/mL phycoerythrin-labeled monoclonal mouse anti-[human IgE] antibody in BSA Block (antibody Clone 4F4cc). Microspheres were then washed briefly 3x 900 μL with TBS-T and re-suspended in 100 μL of TBS-T for readout in a Luminex^®^ MagPix^®^ instrument.

## Results and discussion

### Preparing PC-Antibodies and PC-Beads for use in PC-PURE: Measurement of IgE capture and photo-release efficiency without concentrating

**Fig 3:**
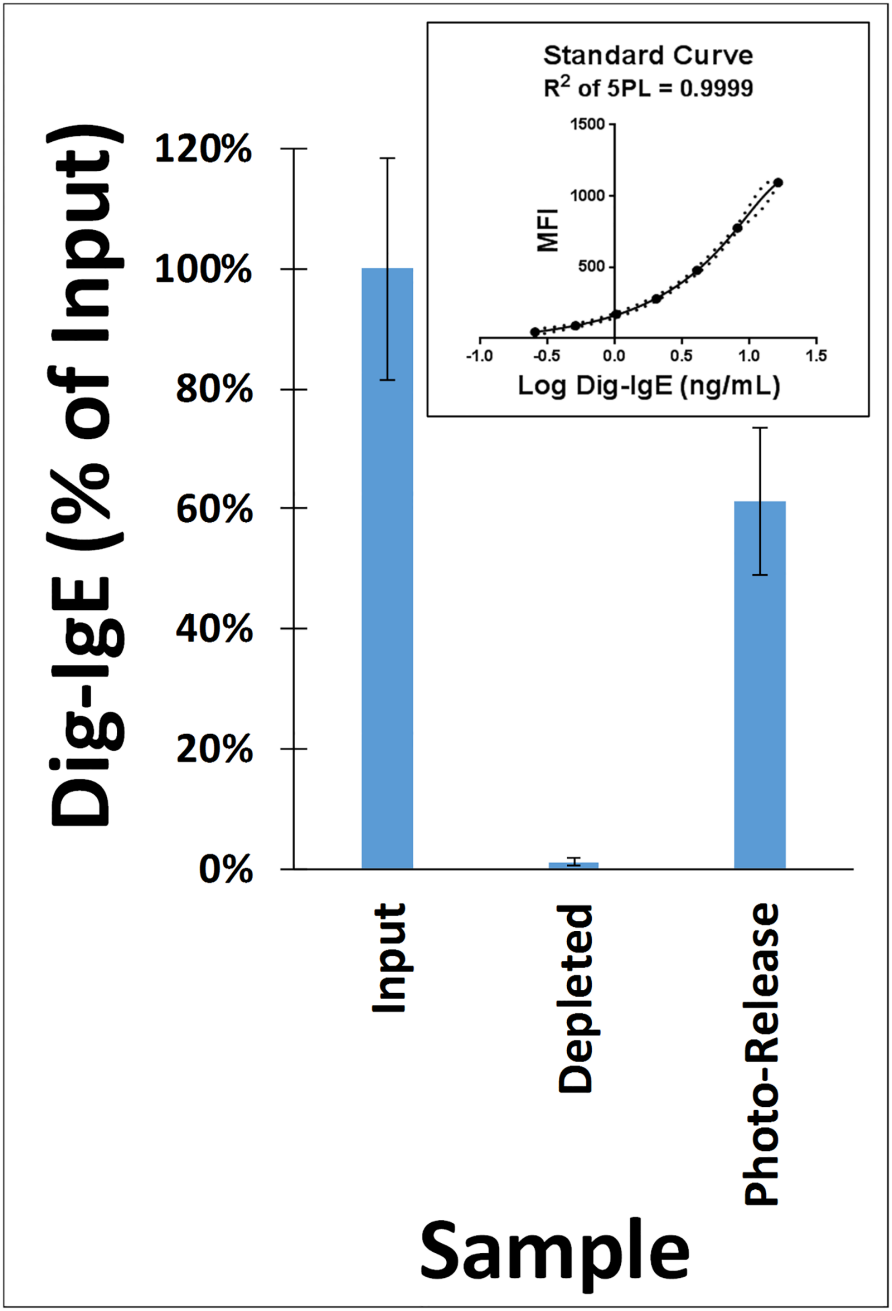
IgE Capture and photo-release efficiency of PC-Beads. Digoxigenin labeled human IgE (Dig-IgE) was captured on PC-Beads which contained the anti-IgE PC-Antibody. PC-Beads were then washed and illuminated with 365 nm UV light. Using a Luminex^®^ based sandwich immunoassay, the amount of Dig-IgE was quantified in the “Input” (solution prior to adding to PC-Beads), “Depleted” fraction (solution after treatment with the PC-Beads) and “Photo-Released” fraction (solution after UV treatment of PC-Beads). For the immunoassay, an anti-digoxigenin capture antibody on the Luminex^®^ microspheres and an anti-IgE detection antibody were used (detection antibody binds different epitope than the PC-Antibody). The immunoassay results were interpolated from a Dig-IgE standard curve using a 5-Parameter Logistic (5PL) curve fit (see inset box; dotted lines are the 95% confidence bands; MFI = Median Fluorescence Intensity of the Luminex^®^ immunoassay). In the bar graph, the amount of Dig-IgE measured is expressed as a percent of the Input (error bars represent the standard deviation of the mean of replicate experiments).

The first step was to prepare an anti-IgE PC-Antibody which was suitable for isolating total IgE prior to its input into the AllerBead assay. For this purpose, a mouse monoclonal anti-IgE antibody (clone E411), which binds the Fc region of human IgE, was labeled with photocleavable biotin (PC-Biotin) to create the PC-Antibody. The PC-Antibody was then loaded at 25 μg per 5 μL of streptavidin agarose bead pellet volume to create the PC-Beads (as detailed later, 5 μL PC-Beads is used for each patient serum sample). Binding assays shown in S1 Fig (Supporting Information) indicate that 99.7% of the added PC-Antibody was bound by the streptavidin agarose beads. Next, to measure the IgE binding and photo-release capability of the PC-Beads, a digoxigenin labeled human IgE tracer was prepared (Dig-IgE). The digoxigenin moiety conjugated to the IgE provided a convenient affinity tag to allow quantification of the Dig-IgE using a Luminex^®^ microsphere-based sandwich immunoassay, where an anti-digoxigenin antibody-coated microsphere captures the Dig-IgE which is then detected using a fluorescently labeled anti-IgE detection antibody (the detection antibody, which is the same detection antibody used for the AllerBead assays detailed later, binds a different epitope on the IgE than the PC-Antibody). Dig-IgE at 4 ng/mL (~2 kIU_A_/L) in 5% BSA/TBS-T was captured by the PC-Beads (5 μL bead pellet), the PC-Beads then washed and photo-release performed (constant volumes of 100 μL were maintained at every step, therefore, Dig-IgE was captured and photo-released but not concentrated in this case). The amount of Dig-IgE was quantified at each step in the process using the aforementioned sandwich immunoassay, which employed interpolation from a Dig-IgE standard curve. Analyzed were the “Input” (solution prior to adding to PC-Beads), “Depleted” fraction (solution after treatment with PC-Beads) and the “Photo-Released” fraction (solution after UV treatment of PC-Beads). The PC-Bead washes contain negligible amounts (shown later) and therefore were not analyzed in this experiment. Results in Fig 3 show that the PC-Beads depleted (bound) 99% of the added Dig-IgE and 62% of the bound Dig-IgE was recovered in the Photo-Released fraction (*i*.*e*. the photo-release efficiency), for an overall yield of 61% (*i*.*e*. recovered Dig-IgE as a percent of the Input). In our prior published work (targeted to proteomic applications), photo-release of [PC-Antibody]-[Target Protein] complexes from agarose beads and measurement by SDS-PAGE showed a similar albeit somewhat higher photo-release efficiency of 77% [46]. The *apparent* lack of 100% photo-release efficiency observed here (increased UV duration did not improve recovery; data not shown) may actually be, in part, a result of the lower-efficiency binding to the Luminex^®^ microsphere surface of the [Dig-IgE]-[PC-Antibody] complexes (in Photo-Released fraction) versus the Dig-IgE alone (in Input solution and Depleted fraction), thereby underestimating the Dig-IgE amount in the Photo-Released fraction. The photocleaved PC-Antibody (still bound to the Dig-IgE) may also sterically hinder the binding of the detection antibody in the Luminex^®^ microsphere-based sandwich immunoassay, again underestimating the Dig-IgE amount in the Photo-Released fraction. It is also possible that a percent of the [Dig-IgE]-[PC-Antibody] complexes remain tightly and non-specifically bound to the PC-Bead surface, and cannot be photo-released.

While the above results demonstrate the basic function of the PC-Beads to capture and photo-release, they do not estimate the maximum IgE binding capacity of the PC-Beads. The PC-Beads should be able to bind the full complement of patient total IgE, even in the most extreme cases (*e*.*g*. atopy). For example, out of the entire 205-member Boston Children’s Hospital (BCH) allergic patient cohort used in this work (detailed later), when total IgE was measured by ImmunoCAP^®^ (data not shown), 1% of the samples exceeded the ImmunoCAP^®^ quantification limit of 5,000 kIU_A_/L with the median measurement 141 and mean 494 kIU_A_/L. Upper limits of normal are between approximately 150 and 300 kIU_A_/L [47, 48]. In one study on individuals with atopic dermatitis, values ranged as high as 12,000 kIU_A_/L [49]. To estimate the PC-Bead binding capacity, a simple depletion assay was performed whereby 5 μL pellet volume of PC-Beads was used to deplete various known amounts of native human IgE spiked into a 5% BSA/TBS-T solution. Similar to the procedure described earlier, the Input solution and Depleted fraction were collected and IgE was quantified (in this case using a standard colorimetric human IgE ELISA). Washes from the PC-Beads after IgE capture were also quantified and found to contain less than 3% (in all washes combined) of the total IgE added. The “Un-Captured” IgE amount was considered as the sum of IgE in the Depleted fraction and all washes. Results shown in S2 Fig (Supporting Information) indicate that the PC-Beads capture 99%, 94% and 74% of the IgE from 5 μg/mL (~2,000 kIU_A_/L), 50 μg/mL (~20,000 kIU_A_/L) and 250 μg/mL (~100,000 kIU_A_/L) solutions, respectively (at 100 μL volume, this is 0.5, 5 and 19 μg of IgE captured; note 2.4 μg = 1 kIU_A_). This demonstrates that sufficient capacity exists to bind the full complement of patient total IgE, even in the most severe cases. Furthermore, the PC-Antibody was highly efficient, with 5 μL PC-Beads containing 25 μg of anti-IgE PC-Antibody able to capture up to 19 μg of total IgE.

### PC-PURE for eliminating the matrix effect with *in vitro* allergy assays

In order to test the ability of PC-PURE to eliminate the matrix effect, we used it as the “front-end” for a multiplex blood-based allergy immunoassay developed by AmberGen, Inc., termed the AllerBead assay, which is based on the Luminex^®^ coded microsphere platform. The overall combined process is illustrated in Fig 2 and consists of the following steps: 1) The blood sample is collected and converted to serum or plasma; 2) Total IgE from the serum or plasma is then captured by an anti-IgE photocleavable antibody (PC-Antibody) immobilized on agarose beads (PC-Beads); 3) The PC-Beads are then washed in microtiter filter plates with a controlled buffer solution to remove interfering sample matrix constituents; 4) The [PC-Antibody]-[IgE] complexes are then gently photo-released in minutes from the PC-Beads using a near-UV light (365 nm); 5) The purified photo-released complexes are re-captured on the multiplex assay surface (Luminex^®^ microspheres in this work) which is coated with specific allergen extracts or allergen component proteins to bind sIgE; 6) The assay is read (Luminex^®^ MagPix^®^ instrument in this work) for detection and quantification. Note that sIgE detection on the Luminex^®^ microsphere (not depicted in Fig 2) is through a separate anti-IgE antibody (labeled with phycoerythrin [PE] in this case), which binds a different epitope on the IgE than the PC-Antibody.

The multiplex AllerBead assay in this study utilized primarily whole food extracts (one extract coated onto a particular coded microsphere), since these extracts provide clinically useful information and are used commonly for non-multiplex *in vitro* allergy testing [34, 45]. The whole food extracts chosen for the multiplex AllerBead assay represented the eight most common food allergens (milk, wheat, soy, peanut, tree nut [cashew], egg [white], fin fish [cod] and shellfish [shrimp]) which account for >90% of all pediatric food allergies [32]. In order to achieve higher analytical sensitivity, two additional allergens besides the whole food extracts were utilized since these allergens exist at low relative abundance in the whole food extracts. These component proteins were Ara h 8 for peanut and lactalbumin (Bos d 4) for milk. Note that ImmunoCAP^®^ in some cases has been reported to supplement their allergen extracts with component proteins for higher analytical sensitivity [50]. In all, the 8 whole food extracts and two component proteins resulted in a 10-plex AllerBead assay.

First, we tested linearity of serum dilution in the AllerBead assay with and without PC-PURE. The same PE-labeled anti-IgE detection antibody was used for both AllerBead assay formats and at the same concentration (as noted earlier, this antibody binds a different epitope on the IgE than the PC-Antibody; while the PC-Antibody is not labeled for detection). S3 Fig (Supporting Information) shows a representative serum dilution series from a patient positive for milk sIgE but negative for soy (as determined using the standard ImmunoCAP^®^ assay). Without PC-PURE, AllerBead shows *apparent* saturation for milk at ~10% up to 100% crude serum (100 μL input to AllerBead), as evidenced by the plateaued signals, with rapidly decreasing signal below 10% crude serum. However, this does not actually reflect a real saturation of the sIgE binding. The Luminex^®^ microspheres are actually saturated by interfering components from the serum (bound to the microspheres but not detected) and not saturated with the target sIgE analyte (which is detected). This is evidenced by the PC-PURE approach which extends the linear range for milk sIgE detection to a much higher signal intensity, approximately 3-fold above the crude serum plateau in this case. Note that with PC-PURE, 200 μL input serum to the PC-Beads and 100 μL photo-release volume for analysis by the AllerBead assay was used, to compensate for the roughly 50% losses upon IgE purification as measured earlier (see later for large-scale serum experiments with PC-PURE but without increased input volume). Regardless of whether the IgE was concentrated or not, that PC-PURE yields linear response extending 3-fold above the *plateaued* signals of the crude serum demonstrates a removal of the matrix effect. For the full serum dilution series, R^2^ of the linear regression (for milk) was 0.99 for AllerBead with PC-PURE, compared to 0.19 for AllerBead without PC-PURE (AllerBead on crude serum). Critically, the matrix effect is not simply eliminated by diluting out the crude serum, since the plateaued signal quickly drops below ~10% serum. This may be attributed to the fact that simply diluting the serum does not change the ratio of interfering agents (matrix constituents) to the target agent (sIgE), while PC-PURE does.

### Large-scale serum studies with PC-PURE and AllerBead: Purifying but not concentrating IgE by PC-PURE

Next, we performed a much larger assessment of the ability of PC-PURE to improve the AllerBead assay by purifying the IgE from serum, but not concentrating the IgE in this case (see later for demonstration of concentrating by PC-PURE). A total of 205 serum samples obtained in collaboration with Boston Children’s Hospital (BCH) were used for this work. The AllerBead 10-plex assay was used to quantitatively measure sIgE concentration from the crude serum and the same assay applied to IgE pre-purified from serum using PC-PURE. In addition, results were compared to the standard (FDA-cleared) ImmunoCAP^®^ test (performed commercially on crude serum by the Phadia Immunology Reference Laboratory [PiRL]). Note that the ImmunoCAP^®^ assay is non-multiplex and was performed for each sample for all 8 whole food extracts. For the AllerBead assay without PC-PURE, 100 μL of serum was used as the input sample volume. For PC-PURE, to ensure in this case that any benefits were strictly from removal of the matrix effects, IgE was isolated from 100 μL of serum and the photo-release volume was also 100 μL, which was input into the subsequent AllerBead assay.

**Fig 4:**
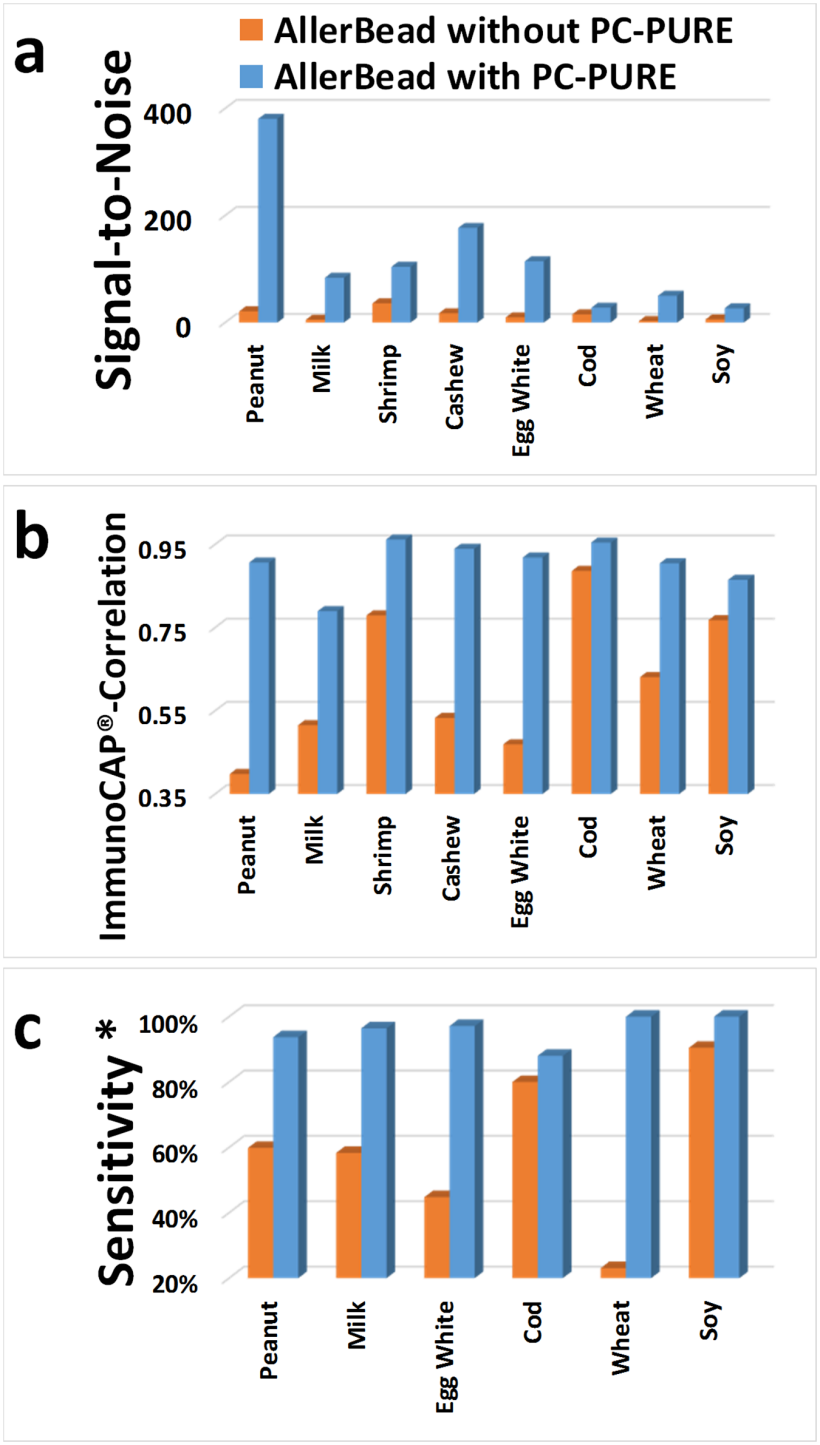
Performance metrics of AllerBead with and without PC-PURE. Serum samples from 205 subjects presenting at Boston Children’s Hospital with suspicion of or known food allergy were analyzed by the multiplex AllerBead assay against all eight food allergens under study. AllerBead was performed with and without PC-PURE. Results from the standard, FDA-cleared, non-multiplex ImmunoCAP^®^ test for all eight foods were used as a reference and to determine true positives and negatives for sIgE. (a) Signal-to-Noise of AllerBead. Signal-to-noise was calculated on a per-food basis as the average AllerBead result for ImmunoCAP^®^-positives (≥0.10 kIU_A_/L) divided by the average AllerBead result of ImmunoCAP^®^-negatives (<0.10 kIU_A_/L). (b) Pearson’s r as a metric for ImmunoCAP^®^-correlation of the AllerBead assays. (c) Sensitivity of the AllerBead assays. *Sensitivity was defined as the percent of ImmunoCAP^®^-positives detected in the range of the maximum measurable by ImmunoCAP^®^ (100 kIU_A_/L) down to the cutoffs for 95% negative predictive value (NPV) for determining clinical allergy. 95% NPV cutoffs ranged from 0.35 kIU_A_/L to 5 kIU_A_/L depending on the food. 95% NPV cutoffs were based on prior literature reports using ImmunoCAP^®^ or equivalent assays in comparison to food challenge (see main manuscript for references); if 95% NPV was not reached in those studies, cutoff for best achieved NPV was used (see Table 1 for cutoffs and NPVs). Note NPV cutoffs have not been published for all eight foods under study and thus Shrimp and Cashew are omitted. AllerBead sensitivity for peanut is a composite of peanut extract and Ara h 8, and for milk, a composite of milk extract and lactalbumin (Bos d 4).

Key results for the AllerBead assay with and without PC-PURE are summarized in Fig 4, including comparisons to ImmunoCAP^®^. AllerBead signal-to-noise (Fig 4a) was markedly improved using PC-PURE, by up to a factor of 18-fold on average for peanut. The smallest increase was a factor 2 on average for cod. Correlation of AllerBead with ImmunoCAP^®^ was determined by Pearson analyses (Fig 4b). Pearson’s r value for AllerBead with PC-PURE averaged 0.90 across the different foods, with all foods ≥0.90 except milk (0.79) and soy (0.86). Pearson hypothesis testing (H_0_: r ≤ 0.5) yielded *p-values* <0.0001 for all foods. In contrast, AllerBead performed without PC-PURE yielded poor ImmunoCAP^®^-correlation, with an average Pearson’s r of 0.62, falling as low as 0.38 for peanut. Furthermore, *p-values* were >0.25 for four foods. To calculate sensitivity (percent of ImmunoCAP^®^-positive patients detected by the AllerBead assays), a scoring cutoff for each food was set at 3 standard deviations above the mean AllerBead result for the ImmunoCAP^®^-negatives (analytical negatives are defined as <0.10 kIU_A_/L by the ImmunoCAP^®^ assay). AllerBead sensitivity (Fig 4c) was defined as the percent of ImmunoCAP^®^-positives detected in the range of the maximum measurable by ImmunoCAP^®^ (100 kIU_A_/L) down to the cutoffs for 95% negative predictive value (NPV) for determining clinical allergy [51–53], since this is the clinically useful range (see Table 1 for details on the NPV cutoffs, which ranged from 0.35 kIU_A_/L to 5 kIU_A_/L depending on the food). Sensitivity of AllerBead with PC-PURE, in this range, averaged 96% for all foods (all ≥94% except soy at 88%). Conversely, sensitivity of AllerBead without PC-PURE averaged only 59%, dropping as low as 23% for wheat.

**Table 1:** AllerBead with PC-PURE in reference to ImmunoCAP^®^ on 205 Boston Children’s Hospital serum samples.

Food	AllerBead Sensitivity to 95% PPV Cutoff^a^ [n, PPV, Cutoff in kIU_A_/L]		AllerBead Sensitivity to 95% NPV Cutoff^a^ [n, NPV, Cutoff in kIU_A_/L]		Min. ImmunoCAP^®^-Positive Detected (ImmunoCAP^®^ kIU_A_/L)	AllerBead Specificity [n]	
Peanut	100%	[75, 100%, 14]	94%	[127, 85%, 0.35]	0.26	95%	[61]
Milk	100%	[48, 95%, 5]	96%	[84, 95%, 0.80]	0.11	94%	[49]
Shrimp	^b^72%	[n = 54]	^b^72%	[n = 54]	0.12	97%	[119]
Cashew	100%	[43, 95%, 15]	^b^93%	[n = 108]	0.23	98%	[83]
Egg White	100%	[74, 95%, 2]	97%	[105, 90%, 0.60]	0.10	98%	[57]
Cod	100%	[7, 100%, 20]	88%	[25, 95%, 0.90]	0.13	97%	[146]
Wheat	100%	[19, 74%, 26]	100%	[39, 95%, 5]	0.11	98%	[63]
Soy	100%	[9, 73%, 30]	100%	[63, 95%, 2]	0.12	97%	[74]

^a^AllerBead sensitivity for detecting ImmunoCAP^®^-positive patients down to the cutoffs for 95% positive and negative predictive value for determining clinical allergy (PPV and NPV; cutoffs listed in table). 95% PPV or NPV cutoffs were from published studies using ImmunoCAP^®^ in comparison to food challenge (see main manuscript for references); if 95% PPV or NPV was not reached in those publications, reported cutoffs for best achieved were used.

^b^PPV and NPV cutoffs not published. In this case, AllerBead sensitivity is listed within the conventional ImmunoCAP^®^ range for analytical-positivity of 0.35–100 kIU_A_/L.

**Fig 5:**
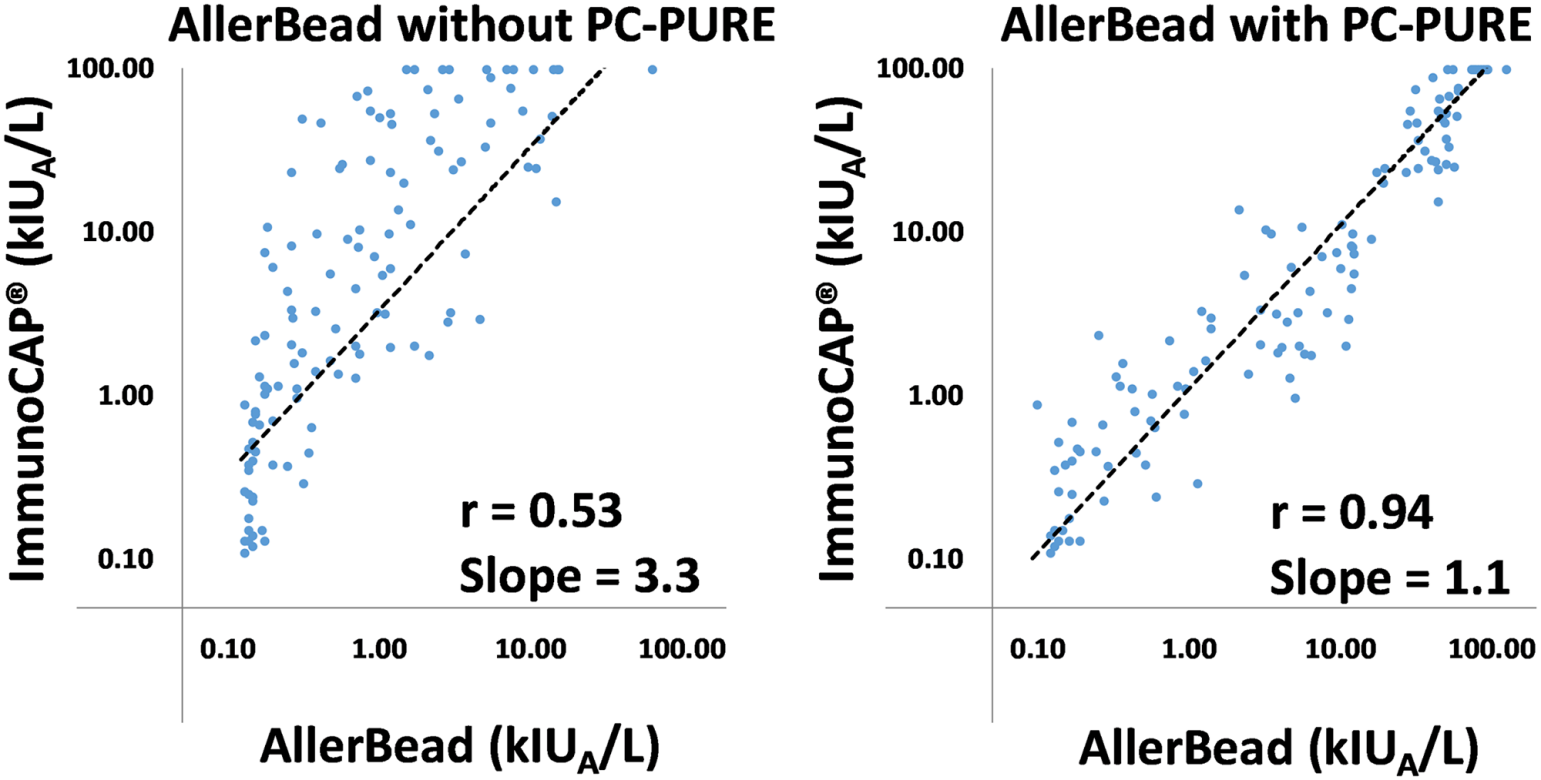
Example ImmunoCAP^®^-correlation of AllerBead with and without PC-PURE. Correlation plot of the multiplex AllerBead assays with and without PC-PURE, compared to the standard, FDA-cleared, non-multiplex ImmunoCAP^®^ test (for the tree nut cashew) for all 205 Boston Children’s Hospital patients. Pearson’s r and slope of the linear regression lines are provided. Note that AllerBead results were converted to kIU_A_/L by heterologous interpolation from a standard curve (5 points; R^2^ of linear regression = 0.99) comprised of purified IgE from the serum of patients with various known amounts of sIgE (based on ImmunoCAP^®^ testing). Pearson’s r for all foods are shown in Fig 4b.

Fig 5 shows a sample correlation plot between AllerBead and ImmunoCAP^®^ for cashew. When PC-PURE was not used for AllerBead, a Pearson’s correlation analysis yields an r value of only 0.53, with the slope of the linear regression line equal to 3.3 (with ImmunoCAP^®^ on y-axis). When PC-PURE was applied to AllerBead, a Pearson’s r value of 0.94 was obtained with the slope of the linear regression line equal to 1.1, indicating an excellent correlation with ImmunoCAP^®^.

Overall, the improvements in AllerBead provided by PC-PURE were achieved despite the fact that the patient IgE was only purified but not concentrated in these particular experiments. The improved signal-to-noise ratio (Fig 4a) was reflected in the improved AllerBead sensitivity for detecting ImmunoCAP^®^-positives (Fig 4c; average 96% for all foods with PC-PURE and 59% without). Critically, the signal-to-noise increase when PC-PURE was used with AllerBead was a result of increased signal, while the background (noise) remained essentially unchanged. The average raw AllerBead result (MFI = Median Fluorescence Intensity) for the ImmunoCAP^®^-negatives (background) in the entire data set (all samples and foods) was 19±15 (±standard deviation) for AllerBead without PC-PURE versus 14±7 with PC-PURE. Conversely, the AllerBead MFI for the ImmunoCAP^®^-positives (signal), when averaged on a per food basis, ranged from 65–675 without PC-PURE and 350–4,637 with PC-PURE, depending on which food. Thus, PC-PURE eliminates signal suppression in the multiplex immunoassay which is caused by the serum matrix. It is important to also emphasize, removal of signal-suppressing agents from the serum was sufficient to overcome the IgE losses incurred by the imperfect photo-release recovery (62% as discussed earlier with the Dig-IgE studies), and therefore PC-PURE was still able provide a significant increase in sensitivity even without concentrating. At least part of the signal suppression caused by the crude (unpurified) serum is expected to be the result of eliminating the competitive binding of non-IgE allergen-specific immunoglobulins (*e*.*g*. IgG and IgA) [54–60]. The data presented here suggests that these and likely other interfering agents from the serum bind and saturate the allergen-coated immunoassay surface and although are not detected, suppress the binding and detection of the target sIgE. This binding capacity problem of multiplex assays is exacerbated especially in allergy testing since the standard practice is to use *whole food extracts* as the antigen on the assay surface (since not all allergenic proteins have been identified). Since whole food extracts can contain hundreds to thousands of proteins, many of which are irrelevant (not allergens), the amount of actual available allergen and hence the surface binding capacity for actual sIgE is *further* reduced. The ImmunoCAP^®^ assay avoids such problems by using an ultra-high capacity cellulose fiber immunoassay surface, which is not readily saturated with interfering agents like the Luminex^®^ microspheres are (see Introduction for further details). However, the ImmunoCAP^®^ approach is not amenable to miniaturization and multiplexing.

Importantly, the aforementioned mode of matrix interference (competition from non-IgE immunoglobulins), and other non-specific modes of the matrix effect (see Fig 1 for possibilities), vary by patient (*i*.*e*. are not a constant). This is shown by the lack of linear correlation with the ImmunoCAP^®^ assay when PC-PURE is not used for AllerBead, in contrast to the excellent linear correlation when PC-PURE is used (see regression plots in Fig 5 for example; Pearson correlation with ImmunoCAP^®^ averages 0.90 for AllerBead with PC-PURE versus 0.61 without; see also Fig 4b for Pearson values per each food).

Finally, Table 1 summarizes additional key figures of merit determined for AllerBead *with* PC-PURE, relative to the standard ImmunoCAP^®^. Of note, AllerBead could detect ImmunoCAP^®^-positives as low as 0.10 to 0.26 kIU_A_/L depending on which food. Sensitivity of AllerBead for all foods was 100% to detect ImmunoCAP^®^-positives in the range of the maximum measurable by ImmunoCAP^®^ (100 kIU_A_/L) down to the cutoffs for 95% positive predictive value (PPV) for determining clinical allergy [51–53], in cases where these cutoffs were available (see Table 1 for further details including the cutoffs, which ranged from 2 kIU_A_/L to 30 kIU_A_/L depending on which food). It should be noted that the AllerBead assay for shrimp showed a lower sensitivity (72% in reference to ImmunoCAP^®^) compared to other allergens (note that no 95% PPV/NPV cutoffs have been reported for shrimp, therefore, AllerBead sensitivity in the conventional ImmunoCAP^®^ reporting range of 0.35 to 100 kIU_A_/L is shown in Table 1). This result is believed to be related to differences in the shrimp species used for the allergen extracts (between AllerBead and ImmunoCAP^®^) and hence differences in representation of the different allergen proteins and isoforms. According to information provided by the manufacturer, the ImmunoCAP^®^ assay for shrimp extract (code f24) uses four species (*Pandalus borealis*, *Penaeus monodon*, *Metapenaeopsis barbata*, *Metapenaeus joyneri*), whereas AllerBead used a commercially available shrimp extract (see Materials and methods) which was comprised of three different species (*Litopenaeus setiferus*, *Farfantepenaeus aztecus* and *Farfantepenaeus duorarum*). Finally, as shown in Table 1, AllerBead specificity was >94% for all foods.

### Using PC-Antibodies to concentrate IgE for the AllerBead assay

In the aforementioned large-scale serum studies, patient total IgE was purified using PC-PURE but not concentrated (100 μL input serum volume and 100 μL photo-release volume). However, an important advantage of the PC-PURE method is the ability to simultaneously concentrate the IgE before the multiplex immunoassay, by inputting a larger serum volume and photo-releasing in a smaller volume than the input serum sample. Importantly, PC-PURE allows the target to be concentrated without concentrating the non-target matrix constituents, and hence the interference which arises from them. This is in contrast to non-specific concentrating methods such as ultra-filtration using molecular weight cutoff membranes. To demonstrate the concentrating abilities, 46 ImmunoCAP^®^-annotated food allergy samples were used. To purify and simultaneously concentrate 5-fold by volume, the PC-PURE input sample volume used was 500 μL, with a photo-release volume of 100 μL. For comparison to the case where no concentration of the sIgE occurs with PC-PURE (purifying only), identical AllerBead measurements were performed on the same samples where the volume input to PC-PURE was 100 μL and the photo-release volume remained the same.

To determine the concentrating efficiency, the raw AllerBead results were used (MFI = Median Fluorescence Intensity) for all sIgE-positives and negatives (sIgE status determined *a priori* by ImmunoCAP^®^ testing). The purifying and simultaneous 5-fold concentrating increased the AllerBead signal 2.7-fold on average compared to purifying only (no concentrating). The fold increase was calculated as the raw AllerBead signal from the 5-fold concentrated samples (average MFI of 1,810) divided by that from the samples which were not concentrated (average MFI of 670), averaged for all known sIgE-positives (determined *a priori* by ImmunoCAP^®^) in the entire dataset for all foods (n = 125). Therefore, the concentrating efficiency was 54%, defined as the observed fold increase in signal by concentrating (2.7-fold) divided by the expected fold increase in signal (5-fold). Importantly, the background (sIgE-negatives; n = 128) was not significantly changed by concentrating (average MFI of 19 for the concentrated samples and 24 for the samples not concentrated).

**Fig 6:**
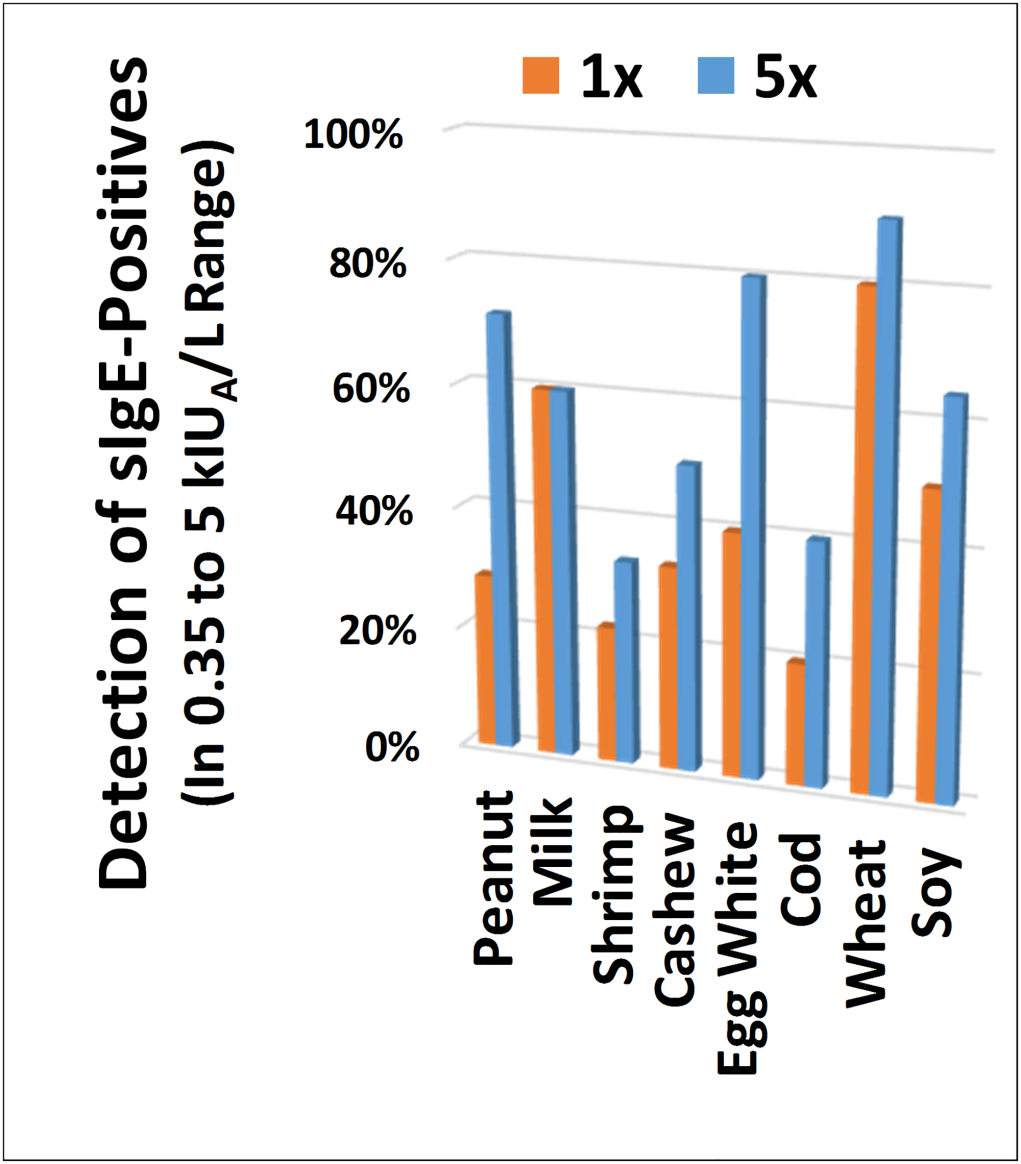
Concentrating patient IgE with PC-PURE: Increased low-end sensitivity for sIgE. PC-PURE was used to simultaneously purify and concentrate IgE from 46 food allergy samples, followed by analysis on the multiplex AllerBead assay. To achieve the concentrating effect, the input sample volume for the PC-PURE step was 500 μL and the photo-release volume was 100 μL (“5x”), which was then input into the AllerBead assay. This was compared to PC-PURE used to only purify but not concentrate the IgE (“1x”; 100 μL input and photo-release volumes). AllerBead sensitivity (percent of sIgE-positives detected) was assessed in the low-end of the ImmunoCAP^®^ scale, defined as between 0.35 kIU_A_/L and 5 kIU_A_/L (sIgE positives determined *a priori* by ImmunoCAP^®^ testing).

Finally, in AllerBead, the most important end-point of concentrating the IgE is detection of low-end sIgE positive samples (low-end sensitivity is important as a negative predictor of clinical allergy [51–53]). Scoring cutoffs for determining AllerBead sensitivity (percent of sIgE-positives detected) were used as described earlier. Fig 6 shows sensitivity in the low-end of the ImmunoCAP^®^ scale (defined as between 0.35 kIU_A_/L and 5 kIU_A_/L). By concentrating, low-end sensitivity of AllerBead was improved for all foods except milk. Most notably, sensitivity improved 3-fold for peanut, and 2-fold each for egg white and cod (overall, this can be attributed to increased signal-to-noise, which in the entire data set improved on average 2 to 4-fold by concentrating, depending on which food; signal-to-noise was calculated as detailed earlier in the legend of Fig 4). The remaining missed detections of sIgE-positives by AllerBead in comparison to ImmunoCAP^®^ are believed in large part to be related to the use of different allergen extract source material between the two assays and the possible lack of or under-representation of certain allergen proteins in the AllerBead assay. However, it should be noted that blood based sIgE testing is notorious for false positives relative to the presence of actual clinical allergy [51–53, 61] (and hence never used alone as a diagnostic), so it is conceivable that PC-PURE employed in AllerBead is providing greater specificity (less false-positive detection) in the low-end compared to ImmunoCAP^®^, although further studies would be necessary to determine this.

## Conclusions

We have developed a new approach, termed PC-PURE, for purification and enrichment of biomarkers in serum and other biofluids which is based on the use of photocleavable antibodies (PC-Antibodies). This “photo-affinity” purification approach is primarily aimed at overcoming the problems caused by the matrix effect which is particularly prevalent for multiplex immunoassays. For example, both false negatives and positives can be produced due to the matrix effect as summarized in Fig 1. Importantly, PC-PURE can also be used to concentrate low-abundance biomarkers, thus enabling more sensitive assays even for non-multiplex measurements. The ability to simultaneously purify and concentrate biomarkers is particularly important since concentration of a biofluid such as serum alone can considerably amplify the matrix effect [29].

In order to evaluate PC-PURE, we applied it initially to the multiplex microsphere-based detection of sIgE from serum. Luminex^®^ MagPlex^®^ magnetic microspheres were configured with allergens specific for a group of the most common food allergies: milk, soy, wheat, egg, peanuts, tree nuts, fin fish and shellfish. This multiplex allergy assay, termed AllerBead, was run on the Luminex^®^ xMAP^®^ platform on 205 serum samples collected from pediatric subjects at Boston Children’s Hospital. AllerBead was performed both with and without the use of PC-PURE to pre-enrich the IgE. The same samples were also analyzed commercially using the standard, FDA-cleared, non-multiplex ImmunoCAP^®^ test. The results demonstrate excellent correlation of AllerBead with ImmunoCAP^®^ when PC-PURE is applied to AllerBead, but poor correlation without PC-PURE (*e*.*g*. average Pearson’s r correlation of 0.90 with PC-PURE vs. 0.61 without, and average sensitivity for detecting ImmunoCAP^®^-positives [down to NPV cutoffs] of 96% with PC-PURE vs. 58% without).

Importantly, PC-PURE eliminates the matrix effect by rapidly pre-purifying the patient IgE into a precisely controlled buffer solution. This contrasts with more conventional approaches of reducing the matrix effect [18, 22, 28, 62, 63], which include custom blocking buffers and diluents specific for each interfering matrix component (*e*.*g*. to block heterophile antibodies), or using selected depletion/inactivation of specific interfering components (*e*.*g*. IgG and IgA in the case of allergy which form part of the matrix effect). These approaches are particularly difficult to apply to complex biosamples such as serum which contain a myriad of interfering agents. Furthermore, in many cases not all interfering components are known and vary by individual [29].

Overall, PC-PURE used as a “front-end” for a multiplex immunoassay can offer several potential benefits including: 1) In contrast to the normal binding of the analyte (*e*.*g*. biomarker) directly onto the immunoassay surface, such as a Luminex^®^ microsphere, PC-PURE provides an additional purification step, enabling more effective removal of interfering matrix components. Ultimately, alternative capture agents can be used for PC-PURE, such as aptamers or protein scaffold based affinity reagents. 2) PC-PURE can utilize a high capacity affinity resin such as the cross-linked porous agarose beads used here. Such resins are widely used in affinity chromatography and are more effective in capturing and purifying the target analyte compared to the low capacity immunoassay surface (*e*.*g*. the solid polystyrene Luminex^®^ microspheres or a planar microarray substrate). Importantly, a high capacity affinity resin more effectively drives the binding kinetics, facilitates more stringent washing and is not readily saturated by interfering matrix contaminants. 3) The highly selective photocleavage-based release of the target biomarker (IgE in this case) leaves behind any matrix agents non-specifically bound to the surface of the affinity resin or substrate. In contrast, elution of the bound target analytes using chemical means including changes in pH, salt, addition of denaturants and chemical cleavage agents can also result in elution of non-specifically bound matrix constituents (along with potentially inactivating the target biomarker). Such additives used for elution may also interfere with the downstream immunoassay.

## Supporting information

S1 Fig Loading the PC-Antibody to streptavidin agarose beads: Preparing PC-Beads. PC-Biotin labeled anti-IgE antibody (PC-Antibody) was loaded onto streptavidin agarose beads to create the PC-Beads. Using a standard commercial colorimetric ELISA, the amount of PC-Antibody was quantified in the “Input” (solution prior to adding to the streptavidin agarose beads) and “Depleted” fraction (solution after treatment with the streptavidin agarose beads). The Blank is the diluent buffer without PC-Antibody. The inset box is the ELISA standard curve using a 5-Parameter Logistic (5PL) curve fit (dotted lines are the 95% confidence bands).
(TIF)

S2 Fig Binding capacity estimate of PC-Beads. PC-Beads carrying the anti-IgE PC-Antibody were used to capture native human IgE spiked at various concentrations into a buffer solution. Using a standard commercial colorimetric human IgE ELISA, the amount of IgE was quantified in the “Input” (solutions prior to adding to the PC-Beads) and “Depleted” fractions (solutions after treatment with the PC-Beads). The IgE in the post-capturing washes was also quantified and summed together with the results from the Depleted fractions; this is reported as the “Un-Captured” IgE amount. *The “Captured” IgE amount is calculated as the difference between the Input and the Un-Captured. The “Blank” corresponds to a Depleted fraction from a 0 μg/mL IgE Input. The inset box shows the ELISA standard curve with a 4-Parameter Logistic (4PL) curve fit.
(TIF)

S3 Fig Elimination of the matrix effect from AllerBead assays using PC-PURE. Multiplex AllerBead assays were performed with and without PC-PURE (which pre-purifies patient IgE). A model patient serum was used for this analysis which was known to be positive for milk sIgE and negative for soy (determined *a priori* based on the standard, FDA-cleared, non-multiplex ImmunoCAP^®^ test). MFI = Median Fluorescence Intensity output of the Luminex^®^ based AllerBead assays.
(TIF)
