# Supplementary figures and images for: Photocleavage-based affinity purification of biomarkers from serum: Application to multiplex allergy testing
Source: PLoS One. 2018 Feb 1;13(2):e0191987. doi: 10.1371/journal.pone.0191987 (PMC5794080; doi:10.1371/journal.pone.0191987)

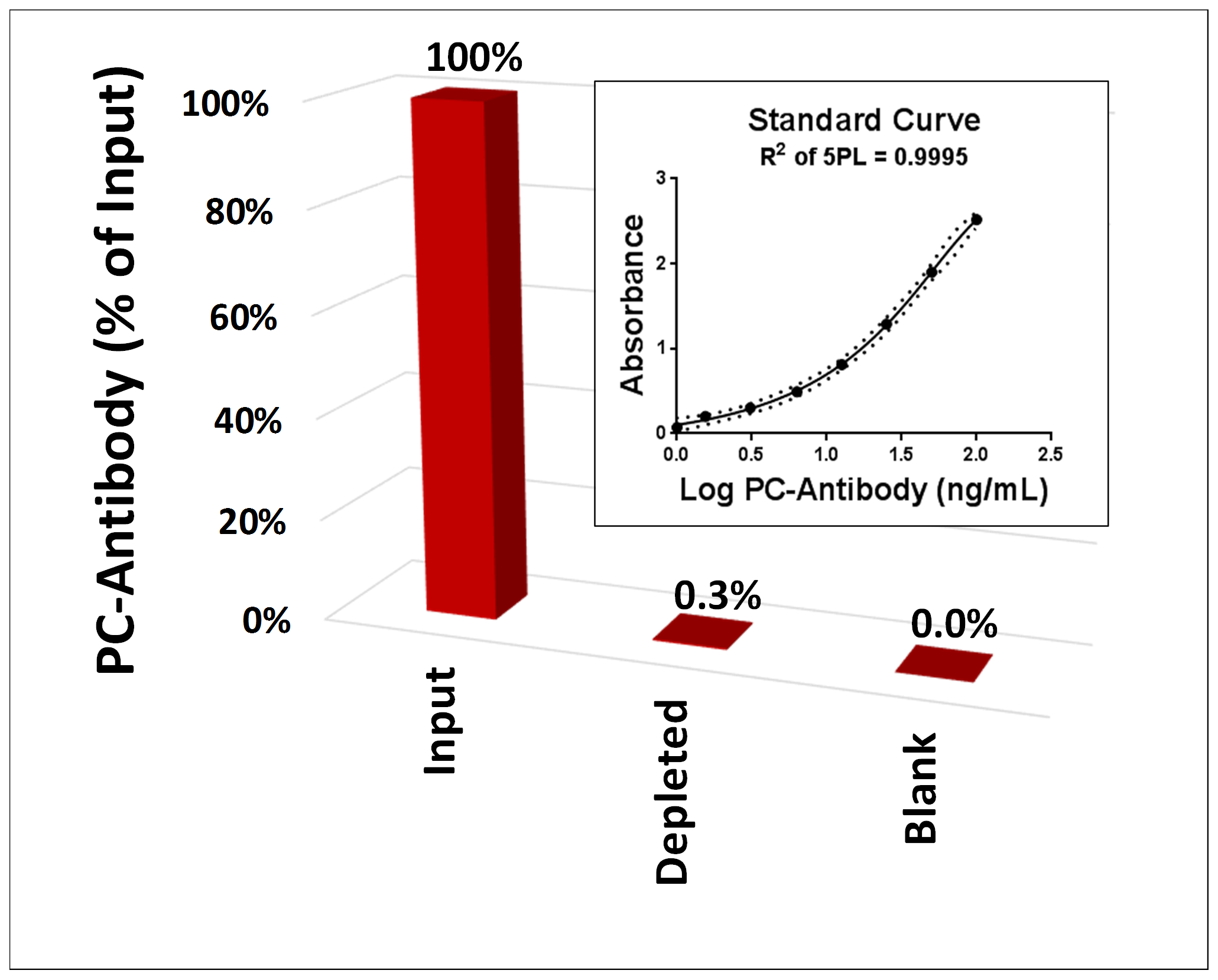

Supplement: S1 Fig — PC-Biotin labeled anti-IgE antibody (PC-Antibody) was loaded onto streptavidin agarose beads to create the PC-Beads. Using a standard commercial colorimetric ELISA, the amount of PC-Antibody was quantified in the “Input” (solution prior to adding to the streptavidin agarose beads) and “Depleted” fraction (solution after treatment with the streptavidin agarose beads). The Blank is the diluent buffer without PC-Antibody. The inset box is the ELISA standard curve using a 5-Parameter Logistic (5PL) curve fit (dotted lines are the 95% confidence bands). (TIF) [file pone.0191987.s001.TIF]

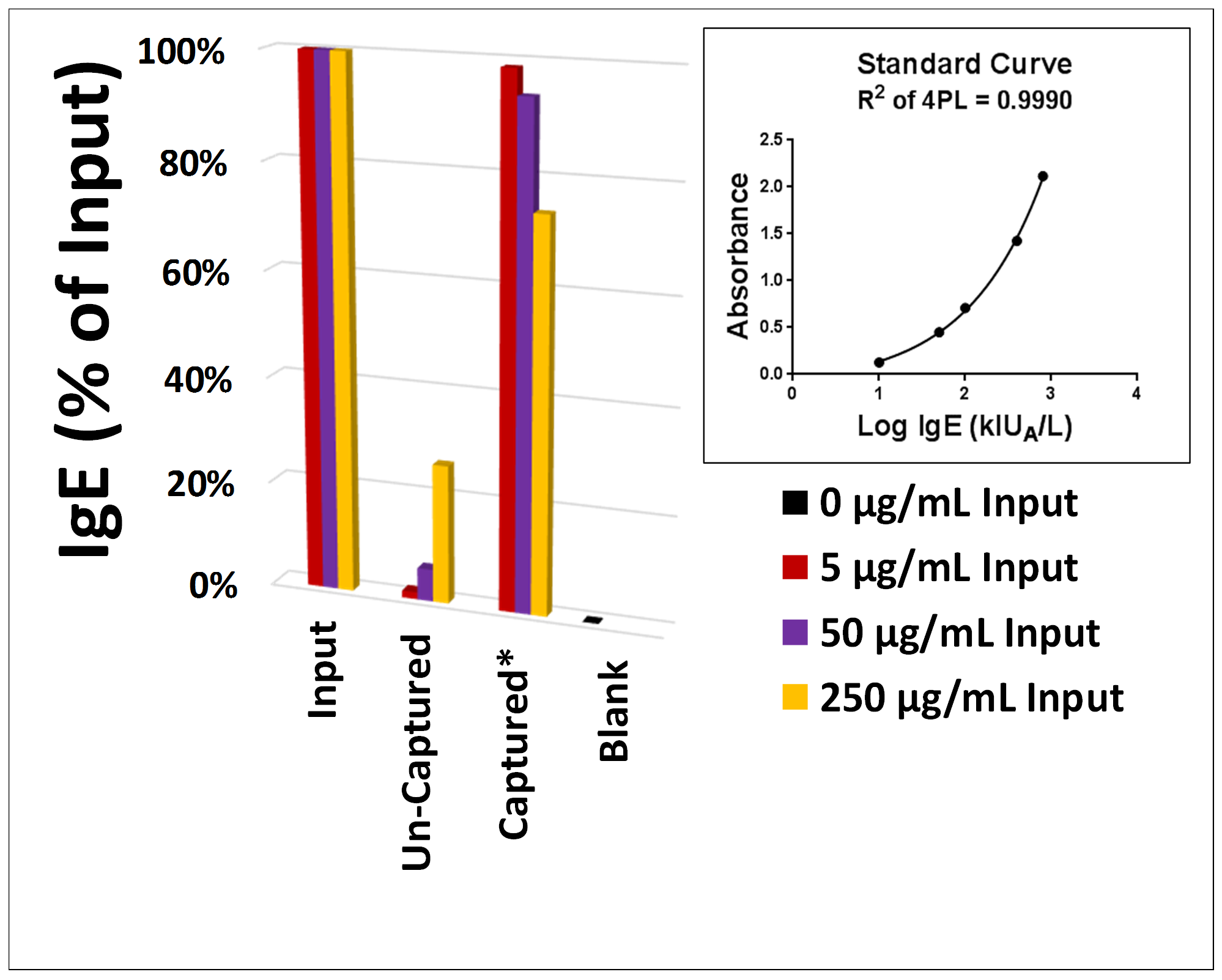

Supplement: S2 Fig — PC-Beads carrying the anti-IgE PC-Antibody were used to capture native human IgE spiked at various concentrations into a buffer solution. Using a standard commercial colorimetric human IgE ELISA, the amount of IgE was quantified in the “Input” (solutions prior to adding to the PC-Beads) and “Depleted” fractions (solutions after treatment with the PC-Beads). The IgE in the post-capturing washes was also quantified and summed together with the results from the Depleted fractions; this is reported as the “Un-Captured” IgE amount. *The “Captured” IgE amount is calculated as the difference between the Input and the Un-Captured. The “Blank” corresponds to a Depleted fraction from a 0 μg/mL IgE Input. The inset box shows the ELISA standard curve with a 4-Parameter Logistic (4PL) curve fit. (TIF) [file pone.0191987.s002.TIF]

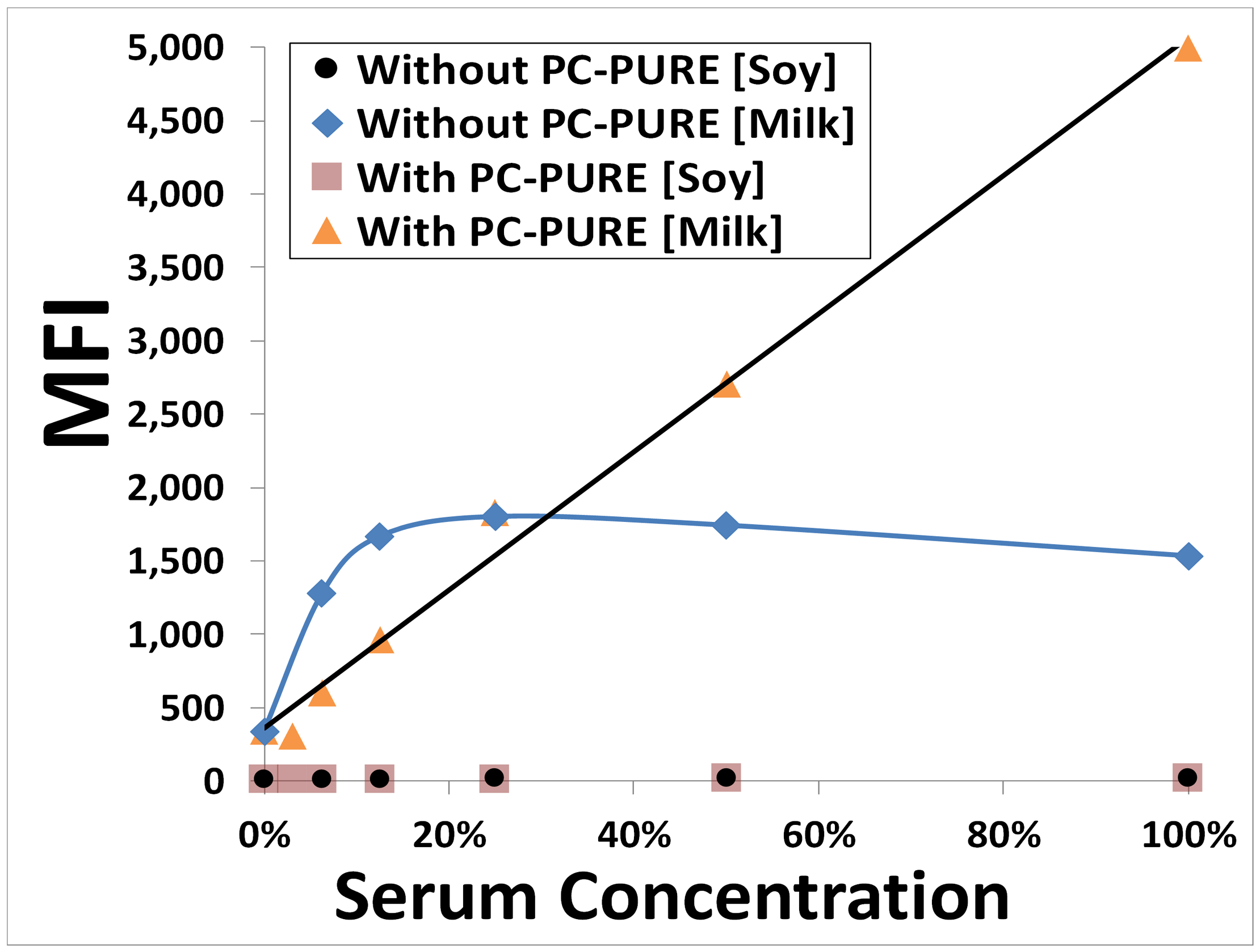

Supplement: S3 Fig — Multiplex AllerBead assays were performed with and without PC-PURE (which pre-purifies patient IgE). A model patient serum was used for this analysis which was known to be positive for milk sIgE and negative for soy (determined a priori based on the standard, FDA-cleared, non-multiplex ImmunoCAP® test). MFI = Median Fluorescence Intensity output of the Luminex® based AllerBead assays. (TIF) [file pone.0191987.s003.TIF]
